# Effects of tourniquet inflation on peri- and postoperative cefuroxime concentrations in bone and tissue

**DOI:** 10.1080/17453674.2021.1942620

**Published:** 2021-08-02

**Authors:** Pelle Hanberg, Mats Bue, Jesper Kabel, Andrea René Jørgensen, Christian Jessen, Kjeld Søballe, Maiken Stilling

**Affiliations:** a Department of Orthopaedic Surgery, Horsens Regional Hospital, Horsens;;; b Aarhus Microdialysis Research Group, Orthopaedic Research Unit, Aarhus University Hospital, Aarhus N;;; c Department of Clinical Medicine, Aarhus University, Aarhus N;;; d Department of Orthopaedic Surgery, Aarhus University Hospital, Aarhus N;;; e Department of Anesthesiology, Horsens Regional Hospital, Horsens, Denmark

## Abstract

Background and purpose — Tourniquet is widely used in orthopedic surgery to reduce intraoperative bleeding and improve visualization. We evaluated the effect of tourniquet application on peri- and postoperative cefuroxime concentrations in subcutaneous tissue, skeletal muscle, calcaneal cancellous bone, and plasma. The primary endpoint was the time for which the free cefuroxime concentration was maintained above the clinical breakpoint minimal inhibitory concentration (T > MIC) for *Staphylococcus aureus* (4 µg/mL).

Patients and methods — 10 patients scheduled for hallux valgus or hallux rigidus surgery were included. Microdialysis catheters were placed for sampling of cefuroxime concentrations bilaterally in subcutaneous tissue, skeletal muscle, and calcaneal cancellous bone. A tourniquet was applied on the thigh of the leg scheduled for surgery (tourniquet duration time [range]: 65 minutes [58–77]). Cefuroxime (1.5 g) was administered intravenously 15 minutes prior to tourniquet inflation, followed by a second dose 6 hours later. Dialysates and venous blood samples were collected for 12 hours.

Results — A cefuroxime concentration of 4 µg/mL was reached within 23 minutes in all compartments and patients. For cefuroxime the T > MIC (4 µg/mL) ranged between 4.8 and 5.4 hours across compartments, with similar results for the tourniquet and non-tourniquet leg. Comparable T > MIC and penetration ratios were found for the first and second dosing intervals.

Interpretation — Administration of cefuroxime (1.5 g) 15 minutes prior to tourniquet inflation is safe in order to achieve tissue concentrations above 4 µg/mL throughout surgery. A tourniquet application time of approximately 1 hour did not affect the cefuroxime tissue penetration in the following dosing interval.

Tourniquet (TQ) is widely used in orthopedic surgery due to its ability to reduce intraoperative bleeding and improve visualization (Rama et al. 2007). However, as the blood supply to the operating field is occluded during surgery, correct timing of antimicrobial prophylaxis administration and TQ inflation is essential in order to ensure therapeutic tissue concentrations at the site of surgery. Only a few studies have investigated the ideal time interval from perioperative antimicrobial prophylaxis administration to TQ inflation, resulting in ambiguous guidelines (Johnson 1987, Deacon et al. 1996, Prokuski 2008, Ochsner et al. 2016). With regard to cefuroxime in particular, a recent randomized controlled microdialysis study in a pig model suggested that a window of 15–45 minutes between cefuroxime administration and TQ inflation results in sufficient perioperative tissue concentrations throughout a 90-minute TQ application (Hanberg et al. 2020b).

TQ induces peri- and postoperative ischemia (Ejaz et al. 2015), which may result in decreased postoperative tissue perfusion and antimicrobial tissue concentration (Smith and Hing 2010). A recent study on a rat model demonstrated a reduced distribution of antimicrobials to TQ-affected tissues for up to 72 hours after TQ release (Mangum et al. 2019). Decreased postoperative antimicrobial tissue concentrations may ultimately increase the risk of surgical site infection.

Therefore, we dynamically evaluated effects of TQ application on both peri- and postoperative in situ cefuroxime concentrations in subcutaneous tissue, skeletal muscle, calcaneal cancellous bone, and plasma. Cefuroxime (1.5 g) was administered intravenously prior to TQ inflation and followed by a subsequent dose 6 hours later. The primary aim was to assess the time for which the free drug concentration of cefuroxime was maintained above the clinical breakpoint minimal inhibitory concentration (T > MIC) for *Staphylococcus aureus* (4 µg/mL) (EUCAST 2021), which we hypothesized was maintained throughout surgery in the TQ-exposed tissues when administering cefuroxime 15 minutes prior to tourniquet inflation.

## Patients and methods

This study was conducted at the Department of Orthopedic Surgery, Horsens Regional Hospital, Denmark. Chemical analyses were performed at the Department of Clinical Biochemistry, Aarhus University Hospital, Denmark. This study was performed in the same setting as another study, which investigated tissue ischemic metabolites (Hanberg et al. 2021).

### Study procedure

#### Microdialysis

The microdialysis catheter consists of a semipermeable membrane at the tip of the catheter, which allows for sampling of water-soluble molecules such as antimicrobials (Hanberg et al. 2016, Kho et al. 2017, Bue et al. 2018, Hanberg et al. 2019b). However, as the semipermeable membrane is continuously perfused, equilibrium across the semipermeable membrane cannot be attained. Consequently, the dialysates represent only a fraction of the actual tissue concentration. This fraction is referred to as the relative recovery that can be determined by different calibration methods (Kho et al. 2017). For this study, meropenem was used as an internal calibrator for cefuroxime (Hanberg et al. 2019a). An in-depth description of the microdialysis technique and the equation for calculating the relative recovery can be found elsewhere (Kho et al. 2017).

We used microdialysis equipment from M Dialysis AB (Stockholm, Sweden). The microdialysis catheters consisted of CMA 63 membranes and CMA 107 precision pumps (flow rate: 2 µL/min).

#### Study design and patients

**Figure 1.**
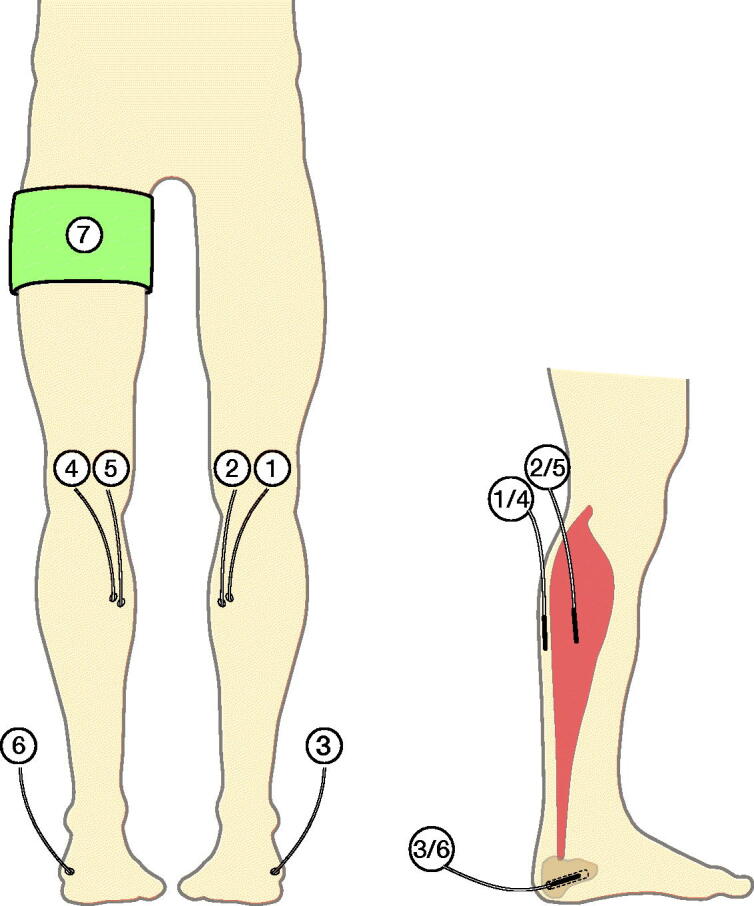
Illustration of the inserted microdialysis catheters. Cefuroxime concentrations were obtained by means of microdialysis catheters placed in non-tourniquet subcutaneous tissue (1), non-tourniquet skeletal muscle (2), non-tourniquet calcaneal cancellous bone (3), tourniquet subcutaneous tissue (4), tourniquet skeletal muscle (5), and tourniquet calcaneal cancellous bone (6). A tourniquet cuff (7) was placed on the leg scheduled for surgery.

10 patients were included in this prospective observational cohort study. The effects of TQ application on both peri- and postoperative cefuroxime concentrations were evaluated in subcutaneous tissue, skeletal muscle, and calcaneal cancellous bone in a simultaneous paired comparison of the TQ and non-TQ leg during 12 hours of continuous microdialysis sampling (Figure 1).

Patients scheduled for hallux valgus or hallux rigidus surgery were offered enrolment in the study. A single surgeon recruited 10 patients who attended the outpatient clinic. Inclusion and exclusion criteria are presented in Table 1. All patients invited for enrolment were included in the study and all completed the study.

**Table 1.** Inclusion and exclusion criteria

Inclusion criteria
Written informed consent
Age ≥ 18 years
Normal distal blood pressure bilaterally
Normal creatinine levels
Use of contraception for women of childbearing age
Exclusion criteria
Diabetes
Unsuccessful spinal anesthesia
Allergy
Previous arterial surgery in either of the legs
Previous surgery on either of the calcaneal bones
Previous fracture or bone infection in either of the calcaneal bones

After placement of the 6 microdialysis catheters, cefuroxime (1.5 g) (FreseniusKabi AB, Sweden) was administered intravenously over 10 minutes, marking time 0. 15 minutes after initiation of the cefuroxime administration, the TQ cuff was inflated (pressure 260 mmHg) on the thigh of the leg scheduled for surgery. Prior to TQ inflation, the leg was elevated for 1 minute. The planned surgical procedure was performed after TQ inflation. When the surgical procedure was completed, the TQ cuff was released (mean TQ inflation time [range]: 65 minutes [58–77]). A second dose of 1.5 g cefuroxime was administered at 6 hours.

#### Surgery

Before the surgical procedure, microdialysis catheters were placed similarly in both legs: in the subcutaneous tissue (membrane length 30 mm), at the posterior site of the mid-lower leg, in the gastrocnemius muscle of the medial head (membrane length 30 mm), and in the calcaneal cancellous bone (membrane length 10 mm) via drill holes (ø: 2 mm; depth 30 mm) made on the posterolateral side, aiming at the anteromedial side of the calcaneal bone (Figure 1). After placement of the microdialysis catheters, all catheters were perfused with 0.9% NaCl containing 5 µg/mL meropenem, allowing for continuous calibration with meropenem as an internal calibrator.

#### Sampling procedures

Dialysates were collected from all 6 microdialysis catheters at 15-minute intervals from time 0–30 minutes, at 30-minute intervals from time 30–180 minutes, and at 60-minute intervals from both time 180–240 minutes and time 300–360 minutes. Following administration of the second dose of 1.5 g cefuroxime at time 360 minutes, dialysates were collected at 30-minute intervals from time 360–540 minutes, and at 60-minute intervals from both time 540–600 minutes and time 660–720 minutes. 17 samples from each microdialysis catheter were collected over the 12-hour period. Venous blood samples were collected at the midpoint of the sampling intervals drawn from a peripheral catheter in the cubital vein. After the last sample was collected, all microdialysis catheters were removed.

#### Handling of samples

The venous blood samples were stored at 5 °C for a maximum of 10 hours before being centrifuged at 3,000 g for 10 minutes. The plasma aliquots were then stored at -80 °C until analysis. The dialysate samples were immediately stored at -80 °C until analysis.

#### Quantification of cefuroxime and meropenem concentrations

The concentrations of cefuroxime and meropenem were quantified using a validated ultra-high-performance liquid chromatography assay (Hanberg et al. 2018). Inter-run imprecisions (% coefficients of variation) were 4.7% at 2.5 µg/mL for quantification of cefuroxime and 3.0% at 2.0 µg/mL for quantification of meropenem. The lower limits of quantification were 0.06 µg/mL for cefuroxime and 0.5 µg/mL for meropenem.

#### Pharmacokinetic analysis and statistics

The cefuroxime concentrations of the dialysate were attributed to the midpoint of the sampling intervals. Pharmacokinetic parameters, areas under the concentration-time curves (AUC), peak drug concentration (C_max_), time to C_max_ (T_max_), and T_1/2_, were determined separately for each compartment for each patient by non-compartmental analysis using the pharmacokinetic series of commands in Stata (v. 15.1, StataCorp, College Station, TX, USA). AUC were calculated using the linear up-log down trapezoidal method. The maximum of all the recorded concentrations was defined as C_max_, enabling calculation of T_max_. The T_1/2_ was calculated as ln(2)/λ_eq_, where λ_eq_ is the terminal elimination rate constant estimated by linear regression of the log concentration on time. The AUC_tissue_/AUC_plasma_ ratio was calculated as a measure of tissue penetration. Microsoft Excel (v. 16.16.11, Microsoft Corp, Redmond, WA, USA) was used to estimate the T > MIC (4 µg/mL) using linear interpolation. The pharmacokinetic parameters and T > MIC were calculated separately for both the first (time 0–6 hours) and second (time 6–12 hours) dosing intervals. The pharmacokinetic parameters and T > MIC were obtained in all 7 compartments from the same patient and a mixed model for repeated measurements had compartments as fixed effect and subject identification variable as a random effect was applied. Also, distinct residual variance was assumed within each compartment. The normality of the residuals was estimated using a quantile–quantile plot for the residuals and the homogeneity of the residual variance was checked by plotting residuals vs. best linear unbiased prediction estimates. The normality of the estimated random effects was checked using a quantile–quantile plot of the estimated random effects. The Kenward–Roger approximation method was used for degrees of freedom correction due to the small sample size (Kenward and Roger 1997). The F-test was used to determine the overall comparisons between the compartments and a t-test was used to determine pairwise comparisons. A significance level of 5% was used. Statistical analyses were performed using Stata.

#### Sample size

Sample size calculation indicated 8 patients, based on cefuroxime target tissue concentrations above 4 µg/mL throughout a predicted surgery time of 105 minutes (pre TQ time [15 minutes] + expected TQ time [90 minutes]). With a significance level of 5% and a power of 90%, a sample size calculation comparing 1 mean to the reference value of 105 minutes was performed for T > MIC (4 µg/mL) for plasma (mean [SD] 145 [28] minutes), TQ subcutaneous tissue (mean [SD] 198 [37] minutes), and TQ calcaneal cancellous bone (mean [SD] 208 [43] minutes) (Hanberg et al. 2020b). To accommodate dropout of patients/microdialysis probes, 10 patients were included.

### Ethics, registration, funding, and potential conflicts of interest

The study was approved by the Danish Medicines Agency (EudraCT number 2018-000217-21), the Central Denmark Region Committees on Health Research Ethics (Registration number 1-10-72-47-18), and the Danish Data Protection Agency (Registration number 1-16-02-88-18). The study was registered at www.clinicaltrialsregister.eu (number 2018-000217-21) and conducted in accordance with the Declaration of Helsinki and the ICH Harmonized Tripartite Guideline for Good Clinical Practice. The Good Clinical Practice Unit at Aalborg and Aarhus University Hospitals conducted the mandatory monitoring procedures.

This work was supported by grants from the Health Research Foundation of Central Denmark Region, the Elisabeth og Karl Ejnar Nis-Hansens Mindelegat Foundation, the Laege Sofus Carl Emil Friis og Hustru Olga Doris Friis’ legat Foundation, the Augustinus Foundation, the A. P. Møller Foundation, and the Familien Hede Nielsen Foundation. The funding sources did not have any roles in the investigation, data interpretation, or paper presentation. The authors have no conflicts of interest.

## Results

The patients’ characteristics are presented in Table 2. No adverse events related to the microdialysis technique or cefuroxime infusion occurred.

**Table 2.** Patient characteristics. Values are mean (range) unless otherwise specified

Sex (female/male), n	7/3
Age, years	58 (45–67)
Height, cm	169 (156–185)
Weight, kg	72 (56–89)
BMI	25 (20–33)
Plasma creatinine, µmol/L	75 (60–90)
Tourniquet duration, min	65 (58–77)
Ankle–brachial index tourniquet leg	1.11 (0.90–1.28)
Ankle–brachial index non-tourniquet leg	1.08 (0.91–1.28)

Normal range: Plasma creatinine (males), 60–106 µmol/L; plasma creatinine (females), 45–90 µmol/L; ankle–brachial index, ≥ 0.9.

The mean relative recovery (SD) values were 23% (9) for TQ subcutaneous tissue, 20% (7) for non-TQ subcutaneous tissue, 39% (4) for TQ skeletal muscle, 33% (12) for non-TQ skeletal muscle, 21% (8) for TQ calcaneal cancellous bone, and 19% (7) for non-TQ calcaneal cancellous bone.

### T > MIC

**Figure 2.**
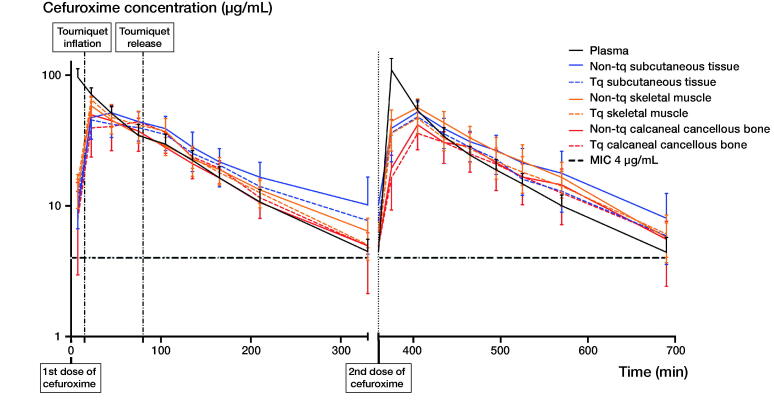
Mean concentration-time profiles of cefuroxime for plasma, subcutaneous tissue, skeletal muscle, and calcaneal cancellous bone on both the tourniquet and non-tourniquet leg. Bars represent 95% CI. The y-axis is in log scale. The first and second dose of 1.5 g cefuroxime was administered at time 0 and 6 h, respectively. Tourniquet inflation and mean release times were 15 and 80 minutes, respectively. Abbreviations: Tq = tourniquet; MIC = minimal inhibitory concentration.

Comparable results were observed for T > MIC (4 µg/mL) between the first and second dosing intervals. Therefore, the T > MIC results are presented only for the first dosing interval in Table 3. A cefuroxime concentration of 4 µg/mL was reached within 23 minutes in all compartments and patients. The T > MIC (4 µg/mL) ranged between 4.8 and 5.4 hours across compartments, and no significant differences were found between the TQ and non-TQ exposed leg (Figure 2 and Table 3). When comparing TQ and non-TQ legs separately, lower T > MIC values were found for calcaneal cancellous bone compared with the remaining compartments in the TQ leg, including plasma (p < 0.05). No differences were found between the compartments in the non-TQ leg.

**Table 3.** Mean time (95% confidence interval) with concentrations above the minimal inhibitory concentration (T > MIC) (4 µg/mL) in minutes for plasma, subcutaneous tissue, skeletal muscle, and calcaneal cancellous bone on both the tourniquet and non-tourniquet leg from the first dosing interval

	Non-tourniquet leg	Tourniquet leg	p-value
Plasma	318 (297–338)	–	–
Subcutaneous tissue	312 (292–333)	322 (302–343)	0.4
Skeletal muscle	320 (300–341)	316 (295–336)	0.7
Calcaneal canc. bone	306 (285–326)	289 (269–310) **^a^**	0.2

**^a^** p < 0.05 for comparison with all compartments on the tourniquet side and with plasma.

### Pharmacokinetic parameters

Comparable pharmacokinetic results were seen between the first and second dosing intervals in all investigated compartments. Only the TQ calcaneal cancellous bone T_max_ was longer in the first dosing interval (mean [range], 84 minutes [23–135]) compared with the second dosing interval (mean [range], 51 minutes [15–75]) (p < 0.01). The pharmacokinetic parameters are presented only for the first dosing interval in Table 4. The concentration time profiles are depicted for both the first and second dosing interval in Figure 2.

**Table 4.** Pharmacokinetic parameters for plasma, subcutaneous tissue, skeletal muscle, and calcaneal cancellous bone on both the tourniquet and non-tourniquet leg

Compartment	Non-tourniquet	Tourniquet	p-value
AUC_0–6h_, mean (95% CI), 10^4^ min µg/mL			
Plasma	8.2 (6.6–9.8)	–	–
Subcutaneous tissue	8.5 (7.0–10)	7.6 (6.0–9.1)	0.3
Skeletal muscle	7.3 (5.7–8.9)	7.8 (6.2–9.4)	0.6
Calcaneal canc. bone	6.6 (5.1–8.2) **^a^**	7.1 (5.6–8.7)	0.6
C_max_, mean (95% CI), µg/mL			
Plasma	97 (84–110) **^b^**	–	–
Subcutaneous tissue	58 (45–70)	51 (38–64)	0.4
Skeletal muscle	61 (48–73)	70 (60–83) **^c^**	0.3
Calcaneal canc. bone	59 (47–72)	53 (40–66)	0.4
T_max_, mean (range), min			
Plasma	7.5 (7.5–7.5) **^b^**	–	–
Subcutaneous tissue	45 (23–75)	49 (23–105)	0.7
Skeletal muscle	27 (23–45)	33 (23–105)	0.5
Calcaneal canc. bone	35 (23–75)	84 (23–135) **^d^**	< 0.01
T_1/2_, mean (95% CI), min			
Plasma	74 (56–93)	–	–
Subcutaneous tissue	94 (75–113)	99 (81–118)	0.7
Skeletal muscle	97 (78–116)	87 (68–105)	0.4
Calcaneal canc. bone	86 (67–105)	95 (77–114)	0.5
AUC_tissue_/AUC_plasma_			
Subcutaneous tissue	1.1 (0.86–1.3)	0.96 (0.73–1.2)	0.3
Skeletal muscle	0.92 (0.69–1.2)	0.98 (0.75–1.2)	0.6
Calcaneal canc. bone	0.84 (0.61–1.1)	0.88 (0.65–1.1)	0.8

AUC, area under the concentration-time curve from 0 to 6 hours; C_max_, peak drug concentration; T_max_, time to C_max_; T_1/2_, half-life; AUC_tissue/_AUC_plasma_, area under the concentration-time curve ratio of tissue/plasma.

**^a^** p = 0.04 for comparison with non-tourniquet subcutaneous tissue.

**^b^** p < 0.05 for comparison with all tissues.

**^c^** p < 0.05 for comparison with tourniquet subcutaneous tissue and calcaneal cancellous bone.

**^d^** p < 0.01 for comparison with tourniquet subcutaneous tissue and skeletal muscle.

No statically significant differences were observed for AUC, C_max_, T_1/2_, and tissue penetrations when comparing the TQ and non-TQ leg (Table 3). Only the calcaneal cancellous bone T_max_ was longer in the TQ leg (mean [range], 84 minutes [23–135]) compared with the non-TQ leg (mean [range], 35 minutes [22–75]) (p < 0.01) in the first dosing interval. No statistically significant differences were found for the remaining compartments.

When comparing the TQ and non-TQ leg separately, a lower AUC was found for the non-TQ calcaneal cancellous bone compared with non-TQ subcutaneous tissue (Table 3). Plasma C_max_ was higher compared with all investigated compartments. Moreover, the TQ skeletal muscle C_max_ was higher compared with both TQ calcaneal cancellous bone and TQ subcutaneous tissue. Finally, plasma T_max_ was shorter compared with all tissues in both the TQ and non-TQ leg, and the TQ calcaneal cancellous bone was longer than both TQ subcutaneous tissue and TQ skeletal muscle.

## Discussion

This is the first clinical study to investigate the effects of TQ application on both peri- and postoperative cefuroxime concentrations in subcutaneous tissue, skeletal muscle, and calcaneal cancellous bone in a simultaneous paired comparison of the TQ and non-TQ leg. Our main finding was a cefuroxime T > MIC (4 µg/mL) range between 4.8 and 5.4 hours across compartments. Furthermore, comparable T > MIC and penetration ratios were found for the first and second dosing intervals.

TQ is widely used in orthopedic surgery, but only a few studies have investigated antimicrobial tissue concentrations during the TQ application, and no clinical studies have investigated antimicrobial tissue concentrations after TQ release. Using bone and fat tissue specimens, Johnson (1987) investigated different time intervals from administration of cefuroxime (1.5 g) to TQ inflation, and concluded that a time interval of 10 minutes was sufficient to achieve tissue concentrations above 4 µg/mL. Recently, a randomized controlled microdialysis study in a pig model suggested that a window of 15–45 minutes between cefuroxime (1.5 g) administration and TQ inflation was sufficient to achieve calcaneal cancellous bone and subcutaneous tissue concentrations above 4 µg/mL (Hanberg et al. 2020b). The present clinical study confirms these findings, suggesting that cefuroxime has fully penetrated the investigated tissues after 15 minutes.

It has previously been hypothesized that perioperative ischemia reduces the postoperative antimicrobial tissue penetration (Smith and Hing 2010, Mangum et al. 2019). However, studies investigating tissue ischemia during and after TQ application found that ischemia-exposed tissue fully recovers 2.5 hours after TQ release (Ejaz et al. 2015, Hanberg et al. 2020b). Our findings do not indicate any decreased postoperative cefuroxime penetration in the TQ exposed tissues for a TQ application of approximately 1 hour.

Interestingly, our study showed that TQ calcaneal cancellous bone T_max_ is longer than in non-TQ calcaneal cancellous bone. Furthermore, a wider range of the T_max_ values was found for both subcutaneous tissue and skeletal muscle in the TQ leg compared with the non-TQ leg. These T_max_ results may be attributed to a combination of the limited elimination rate of cefuroxime during TQ time and a second peak in the cefuroxime concentration after TQ release. For 5 patients, this peak was higher than the initial peak prior to TQ inflation in TQ calcaneal cancellous bone. This may indicate a favorable hyperemic effect when the TQ is released, which was also observed in a pig model (Hanberg et al. 2020b).

For antimicrobial prophylaxis it is generally recommended that the antimicrobial plasma and tissue concentrations exceed the MIC values of relevant bacteria throughout surgery (Mangram et al. 1999). In our study a TQ cuff was inflated 15 minutes after initiation of the cefuroxime administration and a cefuroxime concentration of 4 µg/mL was reached within 23 minutes in all tissues and patients, which was maintained above this target for a minimum of 4.5 hours in all the investigated compartments. As such, these findings indicate that cefuroxime appears to be a good choice for antimicrobial prophylaxis in terms of tissue penetration and T > MIC. Only 1 clinical study has previously investigated cefuroxime bone tissue concentrations by means of microdialysis (Tottrup et al. 2019). Tottrup et al. (2019) found a shorter T > MIC in plasma, subcutaneous tissue, and tibial cancellous bone after a postoperative intravenous bolus administration of 1.5 g cefuroxime compared with our study compartments. While the plasma creatinine was comparable between the patient groups in the 2 studies, Tottrup et al. recorded a substantially higher mean BMI compared with the present study (31 vs. 25). Weight-based dosing of cefuroxime, in addition to consideration of renal function, may therefore be considered in order to achieve therapeutic tissue concentrations in heavy patients.

The few clinical studies that have investigated antimicrobial concentrations during TQ application have been based on tissue specimens (Johnson 1987, Deacon et al. 1996). However, this approach suffers from important methodological limitations because sampling in clinical studies is limited to the time of surgery, free extracellular concentrations cannot be measured selectively, and drug concentrations are given by mass rather than volume (Landersdorfer et al. 2009). Microdialysis, on the other hand, allows for simultaneous and serial sampling of the free and active fraction of drugs in the interstitial space from multiple compartments, both peri- and postoperatively (Tottrup et al. 2016, Hanberg et al. 2020a). These features are desirable, as the majority of infections occur in the interstitial space. However, microdialysis remains a sampling technique that has limitations associated with calibration procedures and chemical assays (Landersdorfer et al. 2009, Kho et al. 2017). The major limitation of our study is the small sample size. Although a paired design and no statistically significant differences for the T > MIC (4 µg/mL) between TQ and non-TQ exposed tissues were demonstrated for this specific patient population, a larger study population may alter these findings. However, as all mean tissue cefuroxime concentrations were above 4 µg/mL approximately 4–5 times longer than the presented surgery time, any potential difference between the TQ and non-TQ exposed tissue may be without clinical relevance.

In summary, administering cefuroxime (1.5 g) 15 minutes prior to TQ inflation seems safe in order to achieve tissue concentrations above 4 µg/mL throughout 1 hour surgery, as T > MIC (4 µg/mL) ranged between 4.8 and 5.4 hours in subcutaneous tissue, skeletal muscle, and calcaneal cancellous bone. A TQ application time of approximately 1 hour did not affect the cefuroxime tissue penetration in the following dosing interval.

PH, MB, JK, KS, and MS initiated and designed the study. PH, JK, and CJ conducted the surgery and placed all the probes. PH, MB and ARJ collected the data. Statistical analysis and interpretation of data was done by PH, MB, JK, KS, and MS. All authors drafted and revised the manuscript.

The authors would like to thank the funding organizations, the Department of Orthopaedic Surgery, Horsens Regional Hospital, and the Orthopaedic Research Unit, Aarhus University Hospital for supporting this study. Finally, they express their sincere gratitude to the patients who participated in this study. 

*Acta* thanks Joan Elizabeth Bechtold and Pim Langendijk for help with peer review of this study.
